# Understanding Human Papillomavirus Vaccine Promotions and Hesitancy in Northern California Through Examining Public Facebook Pages and Groups

**DOI:** 10.3389/fdgth.2021.683090

**Published:** 2021-06-17

**Authors:** Jingwen Zhang, Haoning Xue, Christopher Calabrese, Huiling Chen, Julie H. T. Dang

**Affiliations:** ^1^Department of Communication, University of California, Davis, Davis, CA, United States; ^2^Department of Public Health Sciences, University of California, Davis, Davis, CA, United States; ^3^UC Davis Comprehensive Cancer Center, University of California, Davis, School of Medicine, Sacramento, CA, United States

**Keywords:** HPV vaccine, vaccine hesitancy, Northern California, Facebook, social media, topic modeling, sentiment analysis

## Abstract

Human papillomavirus (HPV) vaccination coverage among adolescents is lower in rural regions and remains under the 80% coverage goal by Healthy People 2030. Through both sentiment analysis and topic modeling, this research examines how local health agencies and groups in nine Northern California counties promote HPV vaccines through Facebook and how target populations react to promotion posts in comments that elucidate their sentiments and hesitancy toward HPV vaccination. In January 2021, we identified 2,105 public Facebook pages and 1,065 groups related to health within the counties and collected a total of 212 posts and 505 comments related to the HPV vaccine. The posts were published between 2010 and 2021, with the majority (83%) published after 2017. There were large variations of Facebook activities across counties. We categorized four counties with HPV vaccination initiation rates below 40% as low-coverage counties and five counties with rates above 40% as high-coverage counties. In general, low-coverage counties had fewer Facebook activities in comparison to high coverage. Results showed that, on average, comments about the HPV vaccine exhibited more positive emotion, more negative emotion, and more anger than the posts. Overall, thematic topics that emerged from posts centered around awareness and screening of HPV and cervical cancer, STI testing services, information sources, and calls to action for health services. However, comment topics did not correspond to posts and were mostly related to vaccine hesitancy, discussing vaccine risks, safety concerns, and distrust in vaccine science, citing misinformation. When comparing high- versus low-coverage counties, posts expressed similar sentiments; however, comments within high-coverage counties expressed more anger than in low-coverage counties. Comments from both high- and low-coverage counties expressed concerns with vaccine safety, risks, and injury. It is important to note that commenters exchanged information sources and tried to address misinformation themselves. Our results suggest that the promotion of HPV vaccines from public Facebook pages and groups is limited in frequency and content diversity. This illustrates problems with generalized social media vaccination promotion without community tailoring and addressing specific hesitancy concerns. Public health agencies should listen to the thoughts of targeted audiences reflected through comments and design relevant messages to address these concerns for HPV vaccination promotion.

## Introduction

Human papillomavirus (HPV) vaccination can effectively prevent infection from the HPV types that can cause certain cancers, including almost all cases of cervical cancer (1, 2). The U.S. Centers for Disease Control and Prevention (CDC) recommends routine HPV vaccination at age 11 or 12 years (can start as early as 9 years) and for everyone through age 26 years (3, 4). Despite the public health implications of full vaccination coverage, HPV vaccination rates remain below the Healthy People 2030 goal of 80% (5). Among adults aged 18–26, the percentage who ever received one or more doses of the HPV vaccine increased from 22.1% in 2013 to 39.9% in 2018 (6). In 2019, 54.2% of adolescents aged 13–17 were up-to-date with the HPV vaccine series, and 71.5% received at least one dose of the HPV vaccine (7). Furthermore, regional disparities in HPV vaccine uptake have been well-documented in the literature, with rural adolescents having lower HPV vaccination coverage than their urban counterparts (7, 8). The 2019 National Immunization Survey Teen (NIS-Teen) reported that adolescents living in non-metropolitan statistical areas (MSA) had about a 10% lower HPV vaccination coverage compared with adolescents living inside MSA central cities (7).

Systematic reviews attribute low rates of HPV vaccination coverage to a multitude of factors, such as limited parental knowledge and awareness of the HPV vaccine, lack of a provider recommendation, and concerns about the side effects and efficacy of the vaccine (9, 10). Many of these factors are shaped and influenced by online and offline information exposure and communication about the vaccine. Vaccine hesitancy, generally defined as the “delay in acceptance or refusal of vaccination despite the availability of vaccination services,” (11) has been particularly associated with online communications surrounding vaccines. Because the internet and social media make sharing information, narratives, and opinions easy, bypassing traditional checking, and gatekeeping processes, the resulting information environment is in abundance of contradictory and incomplete information (12, 13). There are extant efforts in vaccine promotions through social media by public health groups and institutions, and research evidence has documented the effectiveness of using social media-based interventions for increasing vaccine knowledge and acceptance (14, 15). While, on the contrary, exposure to anti-vaccine information, including misinformation, has been shown to negatively influence vaccination attitudes, and decisions (16, 17). Social media has facilitated the spread of misinformation, and several studies have documented the prevalence of anti-vaccine sentiments (18, 19). The major types of HPV vaccine misinformation include conspiracy theories, unsubstantiated claims, and risk of vaccine injury (20). In addition, technical infrastructures, including social media recommendation algorithms, interaction designs, and social network structures, can create and reinforce anti-vaccine communities (21, 22), leading individuals to be more extreme in their misbeliefs.

Effective pro-vaccination communication on social media is urgently needed to promote HPV vaccination through targeted social media channels to combat growing Internet HPV misinformation. Few research studies have investigated HPV vaccination promotions by local organizations and groups on social media, and even less is known about the dynamics of how social media users respond to different kinds of promotional messages. Recent research has shown that user reactions and comments are likely to deviate from the purposes of the original messages, and their different opinions can influence the opinions of other viewers toward vaccines (23). Thus, it is important to examine the distributions of user engagements to targeted public vaccine promotion messages.

This research examines how local health agencies and groups in nine counties of Northern California (i.e., Alpine, Amador, El Dorado, Merced, Nevada, Placer, San Joaquin, Stanislaus, and Yolo counties) promote HPV vaccines through Facebook. These nine counties were selected because HPV vaccination coverage in this region is below the state and U.S. (24), and a previous study revealed a great extent of hesitancy toward HPV vaccine among these communities (25). Through examining location-specific Facebook public pages and groups, we examine how target populations react to promotion posts in comments that reveal their sentiments and their hesitancy in terms of different discussion topics toward HPV vaccination. In addition, we compare how online posts and comment discussions differ between counties with higher vs. lower HPV vaccine initiation rates. Findings have significant implications for guiding the public promotion and communication of HPV vaccination on social media.

## Literature Review

Despite national and state-wide efforts to increase adolescent HPV vaccination coverage to 80% (26), data from the California Immunization Registry (CAIR), the statewide immunization information system, revealed that, in 2018 only, about half of adolescents in California completed the HPV vaccine series by their 13th birthday; and coverage varied greatly by county (26). Previous research found HPV vaccine hesitancy sentiments expressed by agricultural workers, rural communities, and Slavic communities residing in Northern California, and, analogously, CAIR has documented lower HPV vaccination rates in counties with a larger proportion of these communities. For example, based on data from CAIR, only 8.0–23.4% of preteens aged 13 years old in California rural counties have been documented as having completed their HPV vaccine series. Among the counties with the largest agricultural productions, HPV vaccine series completion ranged from 22.7–37.4%, and, among the counties with the largest Slavic communities, HPV vaccination series completion ranged from 28.0–38.2% (USDA) (27). These findings indicate that there is a need to examine why rates are low among some Northern California counties, especially those with communities that have expressed increased HPV vaccine hesitancy. In addition, it is important to understand the perceptions of these communities regarding the HPV vaccine to provide recommendations for health providers and public health professionals to address these disparities in coverage.

To understand perceptions and feelings about HPV vaccinations, traditional methods may be difficult for reaching diverse target audiences, in addition to social desirability biases relating to self-report (28). One approach to reach specific populations, as well as to obtain unobtrusive and naturalistic data, is through examining emergent discussions on social media. Most U.S. adults have used at least one social media platform, and Facebook remains to be one of the most popular social media sites; about 69% of U.S. adults report using Facebook (29). Furthermore, the majority of U.S. adults use Facebook regardless of race, income, and urban/rural residence (29), among which certain demographic factors have been associated with vaccine hesitancy.

For the current research context, previous research showed that Facebook was cited as the most utilized social media platform among the nine California counties, and addressing social media misinformation was identified as a strategy for combating HPV vaccine hesitancy in these communities (25). With the ability to utilize online geolocation tools to pinpoint community public pages and groups, we aim to hone in on examining areas with low vaccination rates. Analyzing the communication dynamics within Facebook groups and pages would enable researchers to examine the attitudes and opinions of users toward HPV vaccination.

Content analysis, using both qualitative and computational quantitative methods, has been conducted to document the content characteristics of social media data. Several studies revealed that users have often expressed negative sentiments over the vaccine, as well as posting and sharing misinformation on social media. For example, Luisi (30) analyzed 6,506 public HPV vaccine-related Facebook posts published within the first decade, following the FDA's first HPV vaccine approval, and found negative sentiments dominated the posts, and negative posts received significantly more user engagements. Furthermore, time effects suggest that few anti-HPV vaccine posts have encouraged more anti-HPV vaccine posts. Kearney et al. (31) analyzed 360 Instagram posts about the HPV vaccine and found a higher proportion of posts were pro-vaccine compared with anti-vaccine. However, anti-vaccine posts were liked significantly more than pro-vaccine posts. Less than 30.0% of the posts came from health-related sources.

While most content analyses are focused on examining social media posts, very few examine the comment sections, which can reflect more of the reactions and opposing thoughts of the target audiences to the posts. The tendency for social media users to express more negative sentiments and engage with anti-vaccine information will likely be observed in the comment section. In many cases, the comments may drive audiences away from understanding and engaging with the promotional messages from the original posts (23). Therefore, one direction to move this line of research forward is to examine the interactions between posts and comments and to document empirically how they diverge in contents regarding the HPV vaccine. Specifically, posts created on Facebook relating to HPV vaccination may elicit emotional comments from the community, and posts may or may not address or influence the discussion agenda of the views and questions of the target audiences about the vaccine. Thus, understanding the sentiments and contents of both posts and comments are important to gain a full picture of how audiences react to information relating to the vaccine. Furthermore, findings can better inform public health professionals on how best to construct messaging for HPV vaccination promotion and for reducing hesitancy toward the vaccine.

In addition to examining the differences between posts and comments, it is also important to understand differences between counties with high or low HPV vaccination coverages. Research suggests that U.S. regions that have expressed negative views about the HPV vaccine on social media, including discussions of misinformation and safety concerns, may have contributed to low HPV vaccination coverage in that region (12). Using similar computational approaches, Zhang et al. (32) demonstrated that the thematic topics discovered from Twitter discussions were significantly associated with vaccination behavioral indicators collected from national surveys. Differences in discussions surrounding the vaccine, as well as differences in strategies to promote the HPV vaccine, may indicate why there are disparities between high- and low-coverage counties. Analyzing the content topics of posts from high-coverage counties may provide insights into constructing effective vaccine promotion strategies. Furthermore, examining the sentiments and the topics of posts and comments within low-coverage counties may inform future messaging interventions to tackle these issues and promote positive attitudes toward the HPV vaccine.

Lastly, another angle to understand the social media space is to examine HPV vaccination promotion efforts across public pages and groups. Public pages are often set up by organizations or institutions to broadcast messages to their audiences. For example, a county public health page may post about an upcoming vaccination clinic. In contrast, public groups are often set up by individuals and act as a group of individuals that discuss issues relating to their own interests. Previous research examining social media posts related to cervical cancer suggests that organizational senders are often more successful in spreading vaccine-promoting information than individual users (33). We thus expect different sentiments and thematic topics across the public pages and groups, given their different motivations and interests in vaccine discussions.

In sum, we address the following research questions. First, how do posts and comments about HPV vaccination differ in terms of sentiments and thematic topics? Second, how do counties with high vs. low HPV vaccination initiation rates differ in posts and comments on dimensions of sentiments and thematic topics? Third, how do public pages and groups differ in posts and comments on dimensions of sentiments and thematic topics? While examining the datasets, we also documented additional observations that may be insightful for understanding local HPV vaccine hesitancy and discussing social media messaging strategies for the target communities.

## Methods

### Data Collection

To systematically trace the HPV vaccine promotions on Facebook in the targeted regions, we designed multiple data searching and collection strategies. The first step was to compile a list of keyword combinations of locations and public health interests to identify location-specific health-relevant public pages and groups where HPV vaccine discussions were likely to occur. On locations, because there are multiple cities and census-designated places located within a county, we considered both county-level and city/place-level searches and compiled a list of 60 location keywords across the nine counties (see Supplementary Material). On public health interests, we used a list of eight keywords (e.g., health, hospital, community clinic, see Supplementary Material). In total, the combinations yielded 480 unique search terms. We performed the searches on Facebook, using a web scraping tool, Selenium Python (34). Then, a team of four trained research assistants screened the relevance of the resulting pages and groups. Pages and groups were excluded if (1) they were private or closed (i.e., not public); (2) they were not related to the specific location (e.g., cities with the same name but in another county or state); (3) their languages were non-English; and (4) they were about pets or animals, but not human (e.g., animal vaccination). This careful screening yielded 2,105 public pages and 1,065 public groups.

Next, we leveraged Facebook's CrowdTangle data monitoring platform to search within the pages and groups for HPV vaccine-related posts (35). We compiled the initial sets of search keywords to be broadly relevant to all vaccines, expecting that some comments may contain HPV vaccine discussions even when the posts did not directly address them. The search terms included 40 keywords, covering vaccines, in general (e.g., vaccine, vaccination, vax, shot), and specific types of vaccines, in particular (e.g., cancer vax, Gardasil, flu shot, MMR vax) (see Supplementary Material for the full list). As expected, a significant number of retrieved posts contained information about other vaccines, especially with a large increase of COVID-19 vaccine posts since December 2020. Therefore, for the focus of the current project, we applied careful human checking on the relevance of the posts to HPV discussion. The four trained research assistants screened the posts and the comments, and the posts were excluded (1) if they were about general vaccination or other specific vaccines but did not mention HPV or the HPV vaccine and (2) if they were about pets or animals, but not humans (e.g., animal vaccination). After irrelevant posts were removed, we retained a total of 212 posts on HPV vaccination and 505 comments. All data searches and collection were conducted in January 2021.

### Statistical Analyses

We first used descriptive statistics to summarize the number of posts and their engagements in terms of comments, likes, and shares across the nine targeted counties. Then, we used the Linguistic Inquiry and Word Count (LIWC) program to analyze the sentiment and specific negative emotions of the posts and comments (36). LIWC is a computerized text analysis tool and has been widely used to examine sentiment, emotions, and psychological and linguistic styles by analyzing word usage (37).

Sentiment and emotions were measured by the percentage of affective lexicons extracted from the texts of each post or comment. We focused on two general sentiment indicators, positive sentiment (indicating the level of positive emotional expressions of the texts), and negative sentiment (indicating the level of negative emotional expressions of the texts), and three specific negative emotions, including anxiety, anger, and sadness, that are commonly represented when contextualizing vaccine hesitancy. Several studies examining social media vaccine contents have used LIWC (38, 39). For example, Faasse et al. (38) used LIWC to compare language usage in pro- and anti-vaccination comments in response to a high-profile Facebook post. The study analyzed 1,489 comments and analyzed similar emotional dimensions, including positive sentiment, anger, and anxiety. Similarly, Himelboim et al. (39) used LIWC to extract positive sentiment and negative emotions, including anger, anxiety, and sadness.

The sentiment and emotion indicators from LIWC range from 0 to 100%. Based on previous studies of social media data, the average levels for positive sentiment and negative sentiment are 5.48 and 2.14, respectively. The average levels for anxiety, anger, and sadness are much lower, i.e., 0.24, 0.75, and 0.43, respectively (36). We calculated the percentage of positive sentiment, negative sentiment, anxiety, anger, and sadness for each post and comment.

We conducted a series of Welch's unequal variance *t*-tests to compare the sentiment and emotion indicators between the sample of posts and the sample of comments. This test is appropriate for comparing samples with unequal sizes and/or variances. Using the same analytical approach, to explore regional variations, we then compared the indicators between the counties with higher vs. lower HPV vaccine initiation rates. Based on the statistics of HPV vaccine initiation rates, we divided the nine counties into two groups, one with five counties with initiation rates above 40% (including Merced, Placer, San Joaquin, Stanislaus, and Yolo) and the other of four counties with initiation rates below 40% (including Alpine, Amador, El Dorado, and Nevada). Last, to explore whether organizational accounts and individual accounts differ in their sentiment and emotion on HPV vaccine discussions, we compared the indicators of posts and comments coming from the public pages that represent organizational accounts vs. the public groups consisting of individual accounts.

After analyzing sentiments, we used topic modeling, a statistical natural language processing approach to identify thematic topics from the datasets. We used Latent Dirichlet Allocation (LDA) (40), a widely used computational tool for finding underlying abstract topics, to identify thematic topics in the posts and the comments. In LDA, each post/comment is modeled as a mixture of topics, and each topic is a probability distribution over words. The LDA algorithm exploits word co-occurrence patterns to discover underlying topics. We used the package gensim in Python (41) to run the topic modeling. We extracted the number of topics based on optimized model perplexity (41). LDA reported the number of topics with keywords and their relative weights contributing to each of the topics. Two authors qualitatively analyzed the prominent keywords and their referent texts to arrive at meaningful interpretations of the latent thematic topics. To address the three research questions, we qualitatively compared differences in latent topics between posts and comments, between counties with higher vs. lower HPV vaccine coverage rates, and between public pages and groups.

## Results

### HPV Vaccination Promotions and Engagements on Facebook Across the Nine Counties

**Figure 1:**
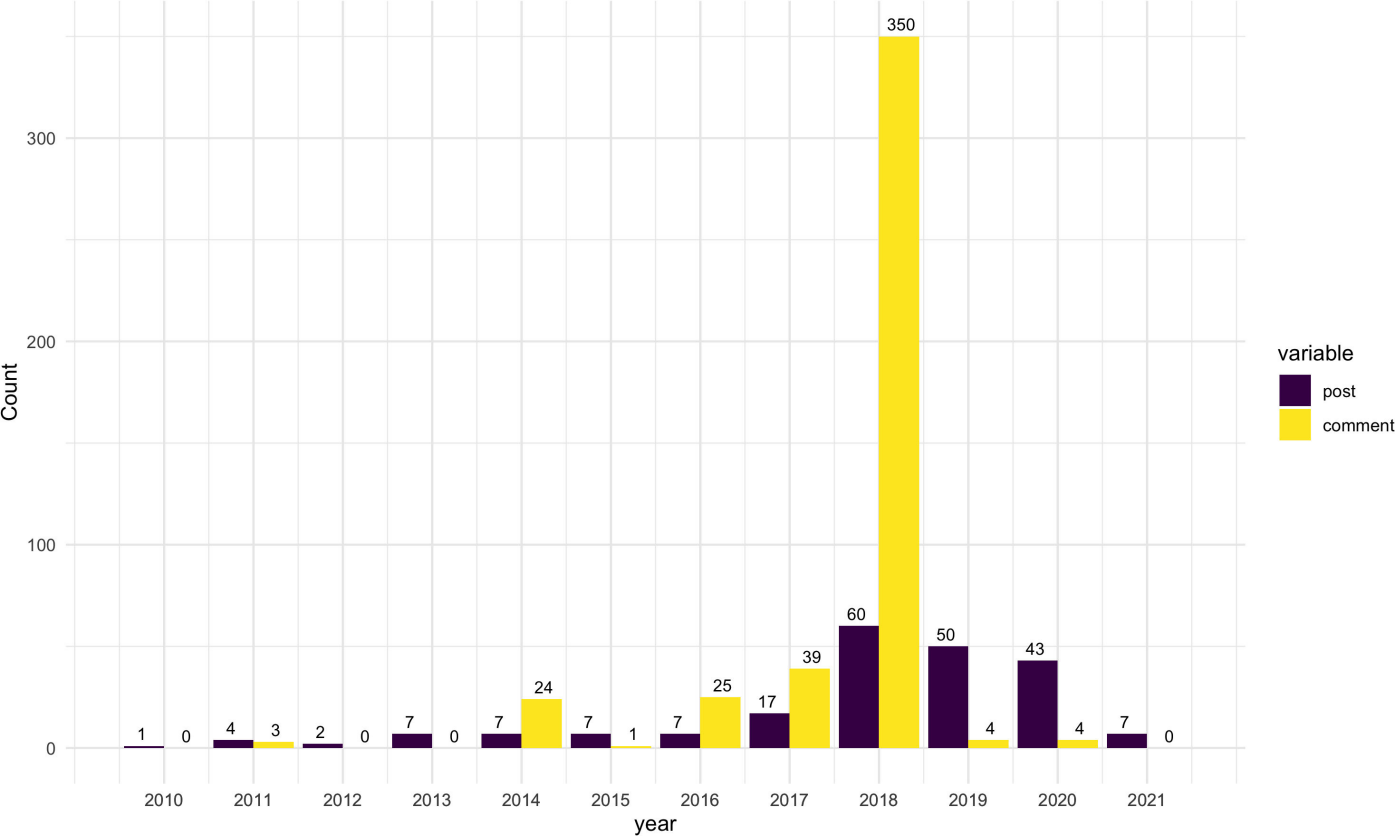
The number of HPV vaccination-related posts and comments on Facebook per year from 2010 to 2021.

We identified more than 3,000 public pages and groups relevant to health discussions in the targeted locations. However, the number of HPV-vaccine-related posts was small. In total, we retrieved 212 posts, with 505 comments, 1,239 likes, and 343 shares. Figure 1 presents the number of posts and comments published over time. The posts were published between 2010 and 2021, with the majority (83%) published after 2017. It is worth noting that a large number of comments were from 2018 due to a highly engaging post from Placer county that generated 334 comments. This post was a standard educational post for the Preteen Vaccine Week, encouraging Placer residents to learn about crucial vaccines to protect their children. Like other similar posts from the identified pages, the post highlighted the HPV vaccine protects against cancer-causing infections for girls and boys. However, the comment section saw a heated debate between the anti-vaccine and pro-vaccine voices and involved sharing and correcting misinformation about the HPV vaccine.

Table 1 presents the summary statistics of posts and their engagements. The majority of posts (208, 98.11%) and comments (450, 89.11%) are from public pages. There were large variations of Facebook activities across the counties. The number of posts ranged from 0 to 71 (M = 26.50, SD = 23.77), and the number of comments ranged from 0 to 338 (M = 2.38, SD = 23.21). Besides, the number of likes was significantly correlated with the number of comments (*r* = 0.21) and shares (*r* = 0.67), and the number of comments and shares was also positively correlated (*r* = 0.35).

**Table 1:** Summary statistics on HPV vaccination rates, the number of Facebook pages, groups, posts, and their engagements across the nine counties.

**County**	**HPV vaccine initiation rate**	**HPV vaccine completion rate**	**Page (*n*)**	**Group (*n*)**	**Post (*n*)**	**Comment (*n*)**	**Like (*n*)**	**Share (*n*)**
Alpine^a^	34.40%	17.30%	36	28	0	0	0	0
Amador^a^	34.40%	17.30%	70	67	2	0	4	0
El Dorado^a^	36.90%	19.60%	251	65	20	17	51	24
Merced	45.30%	22.70%	341	127	18	0	27	9
Nevada^a^	30.90%	15.20%	215	81	10	40	25	3
Placer	49.00%	27.60%	272	131	55	338	85	24
San Joaquin	53.50%	30.30%	289	162	14	2	85	21
Stanislaus	42.20%	21.60%	349	162	22	91	506	120
Yolo	46.20%	28.00%	282	242	71	17	456	142
Total	-	-	2,105	1,065	212	505	1,239	343

a *Counties with underlined HPV vaccine initiation and completion rates were classified as low-coverage counties*.

The lower-coverage counties only accounted for 15.09% of the total posts, 11.29% of the comments, 6.46% of likes, and 7.87% of shares. These suggest that locations with lower vaccination rates also experienced much lower levels of social media promotions and engagements concerning HPV vaccination.

### Sentiments and Negative Emotions in Posts and Comments

Table 2 reports the summaries of average sentiments and negative emotions of posts. Table 3 reports the same statistics regarding comments. We identified significant differences in sentiments in posts and comments. There was significantly more positive sentiment in comments (M = 4.52) than in posts (M = 1.41, *p* < 0.001), more negative sentiment in comments (M = 1.91) than in posts (M =.49, *p* < 0.001), and more anger in comments (M = 0.56) than in posts (M = 0.02, *p* < 0.001). These suggest user comments show higher levels of sentiment overall, both on the positive and negative dimensions. Importantly, the comments also express a significantly higher level of anger.

**Table 2:** Average sentiments and negative emotions of public Facebook posts about the HPV vaccine.

**County**	***N* (212)**	**Positive sentiment**	**Negative sentiment**	**Anxiety**	**Anger**	**Sadness**
		**M (SD)**	**M (SD)**	**M (SD)**	**M (SD)**	**M (SD)**
Amador	2	2.28 (3.22)	0 (0)	0 (0)	0 (0)	0 (0)
El Dorado	20	0.64 (1.33)	0.60 (1.18)	0.25 (0.62)	0 (0)	0.11 (0.40)
Merced	18	2.76 (2.91)	0.31 (1.04)	0.16 (0.67)	0 (0)	0.08 (0.34)
Nevada	10	0.83 (1.75)	0 (0)	0 (0)	0 (0)	0 (0)
Placer	55	1.05 (1.44)	0.61 (1.25)	0.36 (0.90)	0 (0)	0.19 (0.67)
San Joaquin	14	0.94 (1.65)	0.65 (1.49)	0.42 (1.31)	0 (0)	0 (0)
Stanislaus	22	1.79 (3.82)	0.46 (1.32)	0 (0)	0.21 (0.97)	0 (0)
Yolo	71	1.60 (2.26)	0.47 (1.06)	0.34 (0.91)	0 (0)	0.11 (0.55)
Average	26.50	1.41 (2.27)	0.49 (1.15)	0.27 (0.82)	0.02 (0.31)	0.10 (0.49)

**Table 3:** Average sentiments and negative emotions of public Facebook comments about the HPV vaccine.

**County**	***N* (505)**	**Positive sentiment**	**Negative sentiment**	**Anxiety**	**Anger**	**Sadness**
		**M (SD)**	**M (SD)**	**M (SD)**	**M (SD)**	**M (SD)**
El Dorado	17	17.26 (32.19)	0.82 (1.41)	0.17 (0.38)	0.13 (0.45)	0.14 (0.32)
Nevada	40	4.44 (8.21)	2.00 (3.45)	0.09 (0.38)	0.55 (2.25)	0.63 (2.32)
Placer	338	3.11 (5.49)	1.96 (3.16)	0.37 (1.23)	0.54 (1.69)	0.23 (1.53)
San Joaquin	2	0 (0)	0 (0)	0 (0)	0 (0)	0 (0)
Stanislaus	91	6.97 (16.88)	2.26 (6.9)	0.20 (0.82)	0.87 (4.10)	0 (0)
Yolo	17	7.42 (13.31)	0.17 (0.71)	0 (0)	0 (0)	0.17 (0.71)
Average	85.83	4.52 (11.09)	1.91 (4.04)	0.30 (1.08)	0.56 (2.31)	0.21 (1.42)

### Latent Thematic Topics in Posts and Comments

From among all posts, we extracted seven topics. Table 4 summarizes the thematic topics with keywords and associated example post texts. The topics covered overlapping themes with different emphases. Topic 1 centered on the promotion of STI testing for sexually active teens. Example posts called for STI testing and provided detailed information about testing sites. Topics 2 and 5 both revolved around raising awareness of HPV and cervical cancer, especially during January, the Cervical Health Awareness Month. Topic 2 emphasized more on educating women about HPV and cervical cancer, whereas, Topic 5 discussed more about cervical cancer prevention and provided information with frequent references to external websites. Topic 3 emphasized on cervical cancer screening as well but also called on actions for other types of cancer screening. Topic 4 seemed to involve more scientific explanations, aiming to explain disease transmission, and causes with keywords such as disease, transmit, and cause. Topic 6 was characterized by highlighting information sources related to the HPV vaccine, especially referring to government sources, with keywords such as https, gov, and CDC. Finally, Topic 7 aimed for calling for action and directing viewers to clinic locations and health services.

**Table 4:** Thematic topics, keywords, and example Facebook posts about the HPV vaccine.

**Post topic**	**Keywords**	**Example**
1. Promotion of STI testing	HPV, test, https, vaccine, free, parent, STI, www, need, sexually	.There is a teen health clinic at all CommuniCare locations. We offer STI testing, contraceptives and family planning. If you are sexually active, it is time to #GetTested.
2. Awareness on cervical cancer	cervical, health, HPV, cancer, awareness, women, https, month, january, papillomavirus	Cervical cancer was once one of the most common causes of cancer death for American women, but today the death rate is down by more than 50%, thanks to increased cancer screenings and human papilloma virus (HPV) vaccinations. This month, help spread awareness about the importance of the HPV vaccine for cancer prevention.
3. Cancer screening	cancer, HPV, screen, https, neck, vaccine, head, cancers, virus, risk	Do you smoke, drink alcohol, or are sexually active?
		If so, you may be at risk for cancer, and this FREE 10-min screening could save your life.
4. HPV virus and disease transmission	HPV, cancers, https, cancer, papillomavirus, human, vaccine, www, diseases, sexually	Human papillomavirus (HPV) is the most common sexually transmitted infection in the US according to the Centers for Disease Control and Prevention (CDC).
		Researchers report that throat cancers caused by the human papillomavirus, transmitted during oral sex, have increased significantly in the United States.
5. Awareness on cancer prevention	cancer, HPV, cervical, awareness, health, https, month, national, vaccination, risk	Your Cervical Health Begins with Prevention Awareness January is Cervical Health Awareness Month, and CommuniCare Health Centers wants you to know that there's a lot you can do to prevent cervical cancer. [link]
6. Information sources and links	HPV, vaccine, www, cancer, https, unite, gov, prevent, cervical, CDC	To learn more about cervical cancer screenings and the HPV vaccine, schedule a visit with your medical provider or click the following link: https://www.cdc.gov/cancer/cervical/basic_info/screening.htm
7. Call for action	cancer, HPV, cervical, vaccine, virus, test, years, women, oral, cause	January is Cervical Cancer Awareness Month. Have you gotten your Pap or HPV test yet this year? Call to schedule a screening today at a Valley Health Team location near you! Check out the link to learn more: https://www.cdc.gov/cancer/cervical/basic_info/screening.htm
		Did you know? “Two doses of the HPV vaccine are recommended for all boys and girls at ages 11–12; the vaccine can be given as early as age 9.” ~CDC Call Marshall Pediatrics for your vaccinations at (530) 626–1,144

*The topics were ordered by the topic importance calculated with LDA. Keywords included top 10 keywords with the highest weights in a topic, ordered by weights. Examples were partially extracted from representative posts on this topic*.

These seven topics in posts can be thematically categorized into two groups: the first group of posts aims to promote tests, screenings, and vaccination related to cervical cancer, such as Topics 1 (promotion of STI testing), 3 (cancer screening), and 7 (a call for action). The second group of posts aims to introduce more scientific information related to HPV, cervical cancer, and the HPV vaccine, such as Topics 2 (awareness of cervical cancer), 4 (HPV virus and disease transmission), 5 (awareness of cancer prevention), and 6 (information sources and links). The vaccine promotion strategies here are 2-fold: first, to rely mostly on delivering scientific and health information to educate the audiences on the benefits of the HPV vaccine and to raise awareness of cancer prevention and, second, to communicate timely and local service information about testing and vaccination.

Among comments, we also identified seven topics, which are completely different from the post topics. Table 5 summarizes the thematic topics with keywords and associated example post texts. Six out of seven topics related to vaccine hesitancy. Topic 1 mentioned vaccine safety and injuries among children, and a few comments claimed that many pediatricians do not recommend the HPV vaccine, adding references to the VAERS website. Topic 2 was very specific about discussing the HPV vaccine package insert. While we observed anti-vaccine commenters using the texts on vaccine side effects as evidence for vaccine risks and dangers, we also saw comments from pro-vaccine people on correcting the misinterpretation of the insert and asserting vaccine safety. Topic 3 focused on discussing scientific evidence for and against the HPV vaccine, with frequent debates on scientific studies. Some comments also contained multiple external websites and the misinformation that the HPV vaccine is banned in Japan. Topic 4 involved questioning information sources of the people and the validity of the source, with keywords such as source, time, post, and link. Topic 5 was more specific about addressing anti-vaccine claims, with keywords such as kill, insert, report, and article. Some comments mentioned anti-vaccine articles with external links. Topic 6 emphasized vaccine and autoimmune diseases, with keywords of autoimmune, condition, and incidence. Comments discussed whether the HPV vaccine causes autoimmune diseases. Topic 7 was not related to vaccine concerns but rather characterized by frequent keywords used in communication interactions of commenters, such as saying “thank you” or agreeing with the other person with a yes.

**Table 5:** Thematic topics, keywords, and example Facebook comments about the HPV vaccine.

**Comment topic**	**Keywords**	**Examples**
1. Vaccine safety and injuries	vaccine, HPV, know, vaccines, CDC, injuries, children, want, doctor	Even many pediatricians don't recommend this vaccine. Linked to way too many injuries and deaths. Check the CDC and VAERS website. Very irresponsible post Placer County-I'm so saddened by this!
2. Vaccine package inserts	insert, information, vaccine, risk, report, efa, HPV, know, vaccines, CDC	If anyone has read an insert, what is the first line of section 6.1 and 6.2?
		Do you not know what the insert is? It only has premarketing information and it's a legal document. Current studies do prove the safety and efficacy of the vaccine.
		We want to see the insert and that is 100 % accurate and safe.
3. Scientific evidence on vaccines	HPV, https, base, study, vaccines, time, look, shoot, data, science	I understand some people are troubled by science based information. But you do not have a basis to assume others don't want facts and evidence.
		There's plenty of evidence. I personally know a court reporter who has sat in on multiple of these cases. Its everyone's choice obviously, I am just saying do some research before you start giving it to your kids.
		I can't speak to why an Irish politician called to ban a vaccine. But I expect most people understand that is not scientific evidence.
4. Questioning information sources	people, read, vaccines, vaccine, get, HPV, source, time, post, link	Again I ask for where you got your information. I personally have gotten vaccines with a patient information sheet which mentioned POSSIBLE risks. So again, this is false information. What are your sources??
		Cancer is a virus. Read Dr Mary's monkeys some weeks ago I read possible natural cure which was guarantee And I ordered the treatment after 1 week I got 100% cure. I'm so excited to shear this testimony to every article for others living with HPV there is possible natural treatment to eliminate the virus email Dr Onokun, his herbal clinic address.
5. Discussing anti-vaccine information	vaccine, people, cancer, like, source, good, insert, study, HPV, link	Anyone watching the full video mentioned in the article above - from a panel organized by the National Meningitis Association, an organization of parents whose children were killed or disabled by vaccine preventable meningitis - would see the characterization of it in the antivaccine article is incorrect.
6. Vaccine and autoimmune diseases	vaccine, HPV, study, autoimmune, condition, people, group, disease, incidence, receive	Studies show HPV vaccines don't cause autoimmune diseases and paralysis. See my link above.
		Jennifer Robi is a 24-year-old former athlete and scholar who has been confined to a wheelchair since receiving her third Gardasil vaccines at age sixteen. She suffers continual uncontrolled neuro/muscular contractions (jerking) and postural orthostatic tachycardia syndrome (POTS) and many other symptoms of systemic autoimmune dysregulation.
7. User interactions within comment section	vaccine, CDC, read, yes, post, cancer, doctor, HPV, people, know	Thank you for sharing. Your link is terrific. Thanks.

*The topics were ordered by the topic importance, calculated with LDA. Keywords included top 10 keywords with the highest weights in a topic, ordered by weights. Examples were partially extracted from representative posts on this topic*.

Overall, we found the comment section covered prominent discussion topics around vaccine hesitancy. Topics 1 (vaccine safety and injuries), 2 (vaccine package inserts), and 6 (vaccine and autoimmune diseases) were all specifically about safety concerns, citing injuries, and harms. Topics 3 and 4 went beyond specific claims and engaged in more general concerns about scientific research evidence and the study information sources. These two reflect the root skepticism toward science that challenges the confidence of the public toward vaccination. Topic 5 was about addressing anti-vaccine information. While some commenters referred to anti-vaccine articles, others tried to counterargue them. It is interesting to note that Topic 7 reflected on the frequent communication exchanges among the commenters. It is clear that, although some commenters expressed vaccine skepticism or anti-vaccine attitudes, the comment section was not unidimensional, and some commenters were able to confront misleading or false information.

### Differences in Sentiments, Negative Emotions, and Thematic Topics Between High-Coverage and Low-Coverage Counties

As discussed above, we divided the counties into high-coverage counties with an HPV-vaccine initiation rate of over 40%, including Merced, Placer, Stanislaus, San Joaquin, and Yolo, and low-coverage counties, including Alpine, Amador, El Dorado, and Nevada. There were fewer posts on HPV vaccination in low-coverage counties than in high-coverage counties. On average, posts in the low-coverage counties received fewer likes (M = 2.50) than the high-coverage counties (M = 6.44, *p* = 0.01) and, similarly, fewer shares (M = 0.84) than their counterparts (M = 1.76, *p* = 0.03).

Posts and comments in both groups of counties tended to have similar levels of sentiments. For posts, there were no significant differences across all dimensions of sentiment and emotions. However, for comments, there was a significantly higher level of anger in high-coverage counties (M = 0.63) than in low-coverage counties (M = 0.23, *p* = 0.02).

There were differences in the number of themes identified for HPV-related posts and comments in the two groups of counties. For low-coverage counties, there was only one topic in posts and five topics in comments; for high-coverage counties, there were four topics in posts and eight topics in comments (see Supplementary Tables 1, 2). The one topic of posts in low-coverage counties centered on providing HPV-related information and promoting testing. Whereas, the four topics of posts in high-coverage counties covered a more diverse set of information, including awareness of cervical cancer, HPV virus and disease transmission, educational information for women, and a call for action.

Comments in both groups of counties concerned with vaccine safety, risks, and injuries. In low-coverage counties, we extracted five topics. Topic 1 concerned vaccine package inserts, and Topic 3 was on vaccine scientific evidence. Interestingly, we extracted three slightly new topics from this sub dataset. Topic 2 mentioned alternative treatments for cancer, such as promoting natural remedies and downplaying vaccination. Topics 4 and 5 centered on child vaccination, with Topic 5 focus on vaccination especially for boys. These two topics reflect how people debate about the necessity for getting children the HPV vaccine and the confusion or doubts about HPV vaccination for boys. In contrast, the eight topics identified from comments of the high-coverage counties did not refer to specific concerns of children or boys; rather, they emphasized vaccine risks, autoimmune diseases, and questioning of information sources. Interestingly, although, the posts were all about HPV vaccination, some comments digressed to discuss mask-wearing for the COVID pandemic.

### Differences in Sentiments, Negative Emotions, and Thematic Topics Between Public Pages and Public Groups

There were significantly more HPV-related posts by public pages (*N* = 208) than in public groups (*N* = 4). All public page posts were posted by the local government agencies or organizations setting the pages, and all group pages were by individual users. Regarding sentiments, public page posts tended to express more positive emotion (M = 1.43) than group posts (M = 0.17, *p* < 0.001). Besides, public page posts showed significantly more anxiety (M = 0.28) than group posts (M = 0, *p* < 0.001), and more sadness (M = 0.10) than group posts (M = 0, *p* = 0.003). Comments attached to public page posts tended to show more anxiety (M = 0.32) than those for group posts (M = 0.10, *p* = 0.003).

There were four topics identified for page posts and five topics identified for their comments (see Supplementary Table 3). For group posts, there were only two topics for posts and four for comments (see Supplementary Table 4). Public page posts mostly centered on awareness and knowledge promotion, STI testing, and screening. Group posts, in contrast, did not mention anything addressing cancer awareness nor promoting the HPV vaccine but rather digressed to discussing different types of viruses and vaccines, such as mentioning the COVID-19 and SARS in topic 1. Importantly, Topic 2 argued that the Gardasil vaccine caused death.

Given the large data size of comments from the public pages, it is the case that comment topics from the public pages were aligned mostly with the comment topics identified from the overall comment dataset, mentioning vaccine injury, the package inserts, and relevant evidence and information sources. In contrast, comment topics from the groups emphasized vaccine allergic reactions, mask-wearing, HPV vaccine injuries for children, and distrust toward vaccine science.

## Discussion

Social media and Facebook in specific should be used more often to inform and educate the public about HPV vaccination for disease and cancer prevention. Furthermore, given the interactive nature of social media, it is crucial to monitor public sentiments and concerns about the vaccine. As research has advocated for a long time, online health communications cannot just deliver information one-way, assuming that the audiences will accept and be influenced by the messages (14, 42–44). Rather, effective communications need to be two-way interactive so that negative emotions, counterarguments, and concerns of the audiences can be addressed. Social media can afford meaningful asynchronous conversations between the poster and the audience and thus is a potential channel for addressing vaccine hesitancy. As demonstrated by Pedersen and colleagues (14), addressing both cognitive and emotional factors in HPV vaccine hesitancy and devoting resources for community management in terms of creating community dialogues are the keys to restore confidence in HPV vaccination.

Despite the potential, observational accounts of the Facebook public health pages and groups set up within nine counties in northern California do not show adequate two-way communications that respond to the emotional experiences of the target audiences and their specific concerns and worries about HPV vaccination. First, the overall promotion of HPV vaccines from public pages and groups on Facebook is limited in both frequency and content diversity. Most posts focused on general information to raise awareness of cervical cancer, the availability of the HPV vaccine, advocating for HPV vaccination, and direct audiences to external information links. In contrast, the comments did not engage much with such promotional messages and showed significantly higher levels of both positive and negative emotions and, specifically, anger. This finding is in line with a previous study on Twitter data of HPV vaccine conversations, which identified anger from many individual tweets commenting on HPV vaccines (39). Such anger emotion in user comments could further negatively impact the pro-vaccination attitudes of the people (16). This suggests that communication efforts to reduce HPV vaccine hesitancy are needed to strategically address angry reactions of the people.

The topics identified in the comments pertained to discussions about the safety and efficacy of the vaccine (i.e., side effects and reactions) and about HPV vaccine misinformation (e.g., the connection between the HPV vaccine and autoimmune diseases and the banning of the HPV vaccine in other countries), which included further questioning of the information sources and online information sharing attitude of people. In contrast to previous research examining Twitter topics of HPV vaccines that identified a broader spectrum of topics (including conspiracies and policy debates) (45), our data focused on Facebook user comments centered primarily on the direct concerns of the users with side effects and vaccine safety. It suggests that the target audiences of these social media posts do actively participate in the discussions but also try to expand the topics to highlight vaccine hesitancy concerns by exchanging comments. It is also frequent that pro-vaccine people correct misinformation or question the credibility of the information. Here, we need to point out that there is a lack of direct communication by public health pages and group leaders to dispel misinformation and directly address vaccine hesitancy within the comment space.

These observations are best illustrated by the most engaging post from Placer county in our dataset. Although, the post itself was a regular educational message with an attached infographic, it sparked heated debates in its comment section where both pro- and anti-vaccine opinions were expressed, involving sharing, and correcting misinformation about the HPV vaccine. The following two adjacent comments directly illustrated the nature of responses: “Oh great, the anti-vaxxers are out in force tonight. Better go grab my bingo cards.” and “Oh boy, the pro-vaxxers are out in full force tonight. But they don't know HPV vaccine is banned in other counties” (paraphrased quotes). One speculation is that this post included an infographic that might have boosted initial attention from the audience. The other reason may be that Placer county shares a strong conservative base, and vaccine topics incur political concerns. For example, one user commented, “We are a predominantly conservative county and we want to maintain medical freedom, but we are slowly losing it” (paraphrased quotes). This post-comment dynamic is exceptional in the sense that stochastic processes generate very few “black swans” of highly engaged posts in social media (33). However, such post-comment dynamics can be highly influential and provide great insights into vaccine hesitancy. These findings are also in line with another research examining pro- and anti-vaccination comments in response to a high-profile Facebook post, which found that both camps cited external resources and evidence to support their arguments (38).

Furthermore, we observed different patterns in social media promotion and discussion between regions with high vs. low HPV vaccine coverage rates. High-coverage counties devoted more posts to raising awareness and increasing knowledge of the disease causes and transmission and prevention measures, and they also focused on targeting women and directly calling people to take actions for tests and vaccinations. In contrast, low-coverage counties posted more on cancer awareness and general cancer screening. Although, in general, comments from all counties are pertinent to vaccine hesitancy, the differences across the high- and low-coverage counties are important to discuss. Commenters in high-coverage counties focused more on specific topics about vaccine risks and safety concerns (e.g., package inserts and autoimmune diseases), whereas commenters in low-coverage counties discussed more about HPV vaccination for children, especially boys. This might suggest that people in low-coverage counties were mostly concerned with vaccine recommendations for children and may likely be due to the lack of knowledge about how the vaccine works or issues with the false perception that the vaccine encourages sex among youth. Communities in the high-coverage counties likely have higher vaccine acceptance, so most issues pertaining to the HPV vaccine relate to specific misinformation and discussions among those who are still skeptical or anti-vaccine. In perspective of the diffusion of innovation theory (46), this suggests that for counties with fewer adoptions, communicating vaccine recommendations, and explanations of vaccine benefits is more crucial to move people toward accepting the vaccine. In contrast, for high-adoption counties, public communications need to shift to focus on addressing vaccine hesitancy among people who are already aware of the basic information but holding strong misinformed beliefs. This is reflected best in the Placer county case, which contributed 338 comments that included a lot of strong anti-vaccination voices.

Lastly, we observed significantly more public page posts from organizations than group posts by individuals. Page posts and comments were largely focused on the common topics surrounding HPV vaccination promotion or hesitancy concerns, while groups posts and comments sometimes lost focus and digressed to discussing other vaccines for COVID-19 and mask-wearing in general. This suggests that public pages may work more efficiently to have targeted vaccine campaigns for organizations or institutions than setting up public group discussions. Echoing a previous study that found that people are likely to share more organizational messages than individual messages regarding cervical cancer prevention (33), more Facebook public page posts for promoting HPV vaccinations and addressing hesitancy concerns are needed and are expected to be shared more through the social networks of the target audiences.

### Strengths and Limitations

There are several strengths and limitations to be discussed for evaluating the findings. First, in comparison to previous studies that used random sampling of data from social media, this research used precise geolocation searches and rigorous human checking within the platform to zoom into examining the local public pages and groups. Findings from the analyses provide more accurate depictions of the emotional experiences and concerns of the local communities about HPV vaccination. Future research can consider replicating this approach and extend the scope of research to cover more regions and states and explore broader comparisons.

Second, we utilized topic modeling to examine both the posts and comments and illustrated the wide discrepancies in focuses between vaccination promotion messages and reactions of the audiences. The automated modeling approach could increase the comparability of the findings to other analyses, using social media datasets. Given the relatively small sample size, LDA, coupled with qualitative interpretations, provided a high-level extraction of the topics. Future research can further apply the topics to label each post or comment to quantify the percentages of each topic's presence in the data.

We need to point out that this research is exploratory in nature, so we cannot draw clear causal implications of how social media vaccine discussions could impact the target communities. It is also known that social media is not representative of all populations. Given ethical and privacy concerns, we could not extract more information from the individual commenters to describe their demographic backgrounds, such as age, gender, parental status, or race. Knowing this information can help provide a clearer understanding of the active participants and their vaccine stances, future research may consider supplementing social media analyses with surveys.

In addition, while we had two authors qualitatively reading the topic modeling outputs, given the often-fragmented social media comments, we could have under-interpreted or overinterpreted some expressions regarding vaccine hesitancy. The topic modeling results contained overlapping themes, so the demarcation among different topics may not be clear-cut. We attempted to explain the topics with common emphases.

Lastly, we did not harness a lot of data from public groups, since public groups as a feature of Facebook are not popular venues for engaging with health topics. This may be because the most active groups on Facebook are private, and we could not access those. Future research needs to address the challenges of researching private groups, which can provide more insights into vaccine hesitancy in local communities.

## Conclusions and Implications

Public health agencies working for vaccine promotion should expand on social media campaigns and make efforts to improve communications between their page contents and the comments from the targeted audiences. This two-way interactive approach not only leverages the capabilities of social media but allows for an engaged and informed audience in which emotions, concerns, and misinformation surrounding the vaccine can be addressed. Previous research suggests that corrections from reputable sources may help reduce the negative effects of vaccine misinformation (47–49). Public health agencies thus need to first listen to thoughts and misperceptions of targeted audiences reflected through their comments and design relevant messages by citing external expert sources to address the concerns. Formative research to examine concerns about vaccinations in the counties may provide a clear picture for developing future message strategies, a task that is currently being undertaken by our team. This way, agencies can build trust with their communities and foster positive relationships and more effective health communication (44).

Furthermore, public health pages and groups should develop messages that go beyond just providing information and knowledge. This is especially true for the low-coverage counties, where the discussions mostly focus on concerns about the age and gender of administering the vaccine. Developing messages that inform the public on why the HPV vaccine is administered at a younger age, such as how it is more effective when administered before any sexual activity, and messages that address why both boys and girls should get the vaccine, such as how it is used as cancer prevention (and not only cervical cancer), would be particularly useful. In addition, developing more tailored messaging is also important for high-coverage counties because it allows for addressing specific concerns that are brought up as new issues and misinformation are spread. Because emotions are higher among those who exhibit concerns with vaccines, messages can be constructed by leveraging fact-checking labels (49), refutational arguments (16), and narratives that address emotions (50), which have proved to be effective in reducing misinformation impacts.

It is important for public health agencies to examine social media groups and pages within their state and local health department areas to understand the sentiments and contents expressed by their communities. Especially because HPV vaccine skeptic individuals are often within communities that may be hard to reach, public health agencies need to evaluate the roles of social media and allocate resources to their social media communication management. This study provides insights by examining Facebook pages and groups among counties within Northern California. Given that the social media landscape is fast evolving, and young adults and parents under 30 are increasingly using Instagram, Snapchat, and TikTok (29), future research and targeted health promotion campaigns need to examine contents and conversations from these audiences and leverage those platforms for HPV vaccine promotion and communications.

## Data Availability Statement

The raw data supporting the conclusions of this article will be made available by the authors upon request.

## Author Contributions

JZ conceptualized the study. JZ and HX managed the project and conducted thematic interpretations. HX conducted the quantitative analyses and initiated the first draft manuscript with substantial contributions from JZ, CC, and JD. HX, CC, and HC performed data cleaning and coding. All authors contributed to manuscript revision and approved the submitted version.

## Conflict of Interest

The authors declare that the research was conducted in the absence of any commercial or financial relationships that could be construed as a potential conflict of interest.
