# Supplementary material for: Understanding Human Papillomavirus Vaccine Promotions and Hesitancy in Northern California Through Examining Public Facebook Pages and Groups
Source: Front Digit Health. 2021 Jun 17;3:683090. doi: 10.3389/fdgth.2021.683090 (PMC8521881; doi:10.3389/fdgth.2021.683090)
Supplement: Supplementary file 1 [file Data_Sheet_1.docx]

**Supplementary Material**

**Search terms for Facebook pages and groups**

One search term will be a location-based keyword and a health-related keyword, e.g. Alpine community clinic.

| Location-based keywords | Health-related keywords |
| --- | --- |
| Alpine (12):  Alpine, Alpine Village, Bear Valley, Fredericksburg, Kirkwood, Lake Alpine, Loope, Markleeville, Mesa Vista, Paynesville, Sorensens, Woodfords  Amador (6):  Amador, Amador City, Ione, Jackson, Plymouth, Sutter Creek  El Dorado (3):  El Dorado, Placerville, South Lake Tahoe  Merced (7):  Merced, Atwater, Dos Palos, Gustine, Livingston, Los Banos, Merced  Nevada (3):  Nevada, Grass Valley, Nevada City  Placer (6):  Placer, Auburn, Colfax, Lincoln, Rocklin, Roseville  San Joaquin (8):  San Joaquin, Escalon, Lathrop, Lodi, Manteca, Ripon, Stockton, Tracy  Stanislaus (10):  Stanislaus, Ceres, Hughson, Modesto, Newman, Oakdale, Patterson, Riverbank, Turlock, Waterford  Yolo (5):  Yolo, Davis, West Sacramento, Winters, Woodland | Community clinic  Health  Health center  Health department  Health services  Healthcare  Hospital  Public health |

**Search terms for HPV-related posts**

Vaccine, Vaccination, Vax, Vaxx, Shot, HPV, HPV Vax, HPV vaccine, Preteen vaccine, Preteen vax, Preteen shot, Human papillomavirus, Human papillomavirus vax, Human papillomavirus shot, Human papillomavirus vaccine, Cervical cancer vaccine, Cancer shot, Cancer vax, Cancer vaccine, Vaccination for HPV, Gardasil, Gardasil 9, Gardasil vaccine, Gardasil vax, Gardasil shot, Flu vax, Fluvax, Flu vaccine, Flu shot, Nasal flu spray, Influenza vaccine, Tdap vax, Tdap vaccine, Tdap shot, MMR vaccine, MMR vax, MMR shot, Meningitis vaccine, Meningitis vax, Meningitis shot

**Supplementary table 1.** Thematic topics, keywords, and examples Facebook posts and comments about the HPV vaccine in high-coverage counties.

|  | Topic | Keywords | Example |
| --- | --- | --- | --- |
| Post | 1. Awareness on cervical cancer | *hpv, cancer, cancers, cervical, awareness, national, cdc, virus, https, high* | *Your Cervical Health Begins with Prevention Awareness January is Cervical Health Awareness Month, and CommuniCare Health Centers wants you to know...* |
|  | 2. HPV virus and disease transmission | *hpv, cervical, cancer, awareness, cause, test, https, health, need, www* | *HPV is easily avoided and diagnosed with regular check-ups. Take charge of your health this year, and get a check-up today!* |
|  | 3. Educational information for women | *hpv, cervical, cancer, vaccine, https, health, awareness, www, women, papillomavirus* | *Thankfully, young people can get vaccinated against HPV and adult women can get screened at their Pap test.* |
|  | 4. Call for action | *test, hpv, free, cancer, sti, sexually, teen, vaccinations, take, share* | *If you are sexually active, it is time to #GetTested* |
| Comment | 1. Vaccine risks | *vaccine, risk, information, insert, efa, baldwin, learn, like, hpv, fda* | *Again I ask for where you got your information. I personally have gotten vaccines with a patient information sheet which mentioned POSSIBLE risks. So again, this is false information. What are your sources??* |
|  | 2. Vaccine and autoimmune diseases | *vaccine, hpv, study, www, people, https, com, look, autoimmune, incidence* | *Studies show hpv vaccines don't cause autoimmune diseases and paralysis. See my link above.* |
|  | 3.Questioning information sources | *people, read, vaccine, information, donã, source, science, look, understand, report* | *People can read the sources in the post about hpv vaccines. Since it's unclear which information you want a source on, I can't provide one.* |
|  | 4. Scientific evidence on vaccines | *evidence, great, dr, thank, understand, job, try, insert, mahima, visit* | *Also do you understand the hierarchy of evidence in science? This plays a part as well. A comprehensive review is high on the pyramid of evidence.* |
|  | 5. Mask wearing | *mask, wear, cancer, placer, county, dorit, rubinstein, reiss, cause, time* | *Wear a mask after vaccination? So I dont give the virus to my vaccinated grandmother? Seems legit.* |
|  | 6. Vaccine recommendation | *vaccine, hpv, county, cdc, recommend, get, vaccines, placer, know, post* | *Even many pediatricians don't recommend this vaccine. Linked to waaay too many injuries and deaths. Check the CDC and VAERS website. Very irresponsible post Placer County-I'm so saddened by this!* |
|  | 7. Vaccine data | *vaccines, vaccine, data, people, hpv, learn, www, know, court, efa* | *Scientists are working to get data on hpv vaccines. Their results show that these vaccines are safe and effective.* |
|  | 8. Vaccine package inserts | *vaccine, insert, doctor, read, hpv, vaccines, know, time, things, dorit* | *And a new doctor.... please read the info out there. Even my own doctor told me not to bother with this one... and he's been around a heck of a lot longer than you and works for a very large well respected hospital system.* |

**Supplementary table 2.** Thematic topics, keywords, and examples Facebook posts and comments about the HPV vaccine in low-coverage counties.

|  | Topic | Keywords | Example |
| --- | --- | --- | --- |
| Post | 1. HPV information and cancer screening | *hpv, cancer, https, screen, vaccine, risk, cancers, papillomavirus, www, neck* | *Cervical Cancer Screening Made Simple Do I need a Pap? An HPV test? When? Find out: [link]* |
| Comment | 1. Vaccine package inserts | *vaccine, people, vaccines, hpv, teethe, cause, cdc, report, assault, list* | *For example on the chickenpox vaccine insert it lists teething as an adverse event. Things being reported, like teething, happen at the same time, but they do not cause teething.* |
|  | 2. Treatment discussion | *vaccine, hpv, study, com, insert, treatment, include, state, https, cure* | *some weeks ago i read possible natural cure which was guarantee And I ordered the treatment after one week i got 100% cure. I'm so excited to shear this testimony to every article for others living with hpv there is possible natural treatment to eliminate the virus email Dr Onokun* |
|  | 3. Scientific evidence on vaccines | *vaccine, understand, science, information, evidence, look, base, know, people* | *I understand some people are troubled by science based information. But you do not have a basis to assume others don't want facts and evidence.* |
|  | 4. HPV vaccines for children | *know, virus, study, people, vaccine, research, child, cervix, include* | *no evidence to support your claim. I know so many people who have vaccinated their children with this, and are healthy adults now. Trust me, you dont want you or anyone you love getting cervical cancer, I wish they had this vaccine sooner!!* |
|  | 5. HPV vaccines for boys | *vaccine, hpv, cdc, flu, boys, go, health, cancer, kid, link* | *Awesome job..and FYI..boys can get the vaccine as well because boys and men are the carriers of HPV..you can even Google that fact.*  *There's plenty of evidence. I personally know a court reporter who has sat in on multiple of these cases. Its everyone's choice obviously, I am just saying do some research before you start giving it to your kids.* |

**Supplementary table 3.** Thematic topics, keywords, and examples Facebook posts and comments about the HPV vaccine from public pages.

|  | Topic | Keywords | Example |
| --- | --- | --- | --- |
| Post | 1. Cervical cancer awareness promotion | *hpv, cervical, awareness, cancer, month, health, papillomavirus, https, human, january* | *January is Cervical Cancer Awareness Month, a great time to talk about how human papillomavirus (HPV) vaccines can help prevent cancer.* |
|  | 2. Cancer screening | *hpv, cancer, neck, cancers, head, include, screen, https, org, risk* | *Free Oral, Head and Neck Cancer Screening* |
|  | 3. Educational information on HPV and cancer prevention | *hpv, cancer, vaccine, cervical, https, cancers, www, cause, cdc, human* | *Human Papillomavirus (HPV) is a common sexually transmitted infection. A pap smear can help detect changes in the cervix caused by HPV. Learn more.* |
|  | 4. Promotion of STI testing | *test, cervical, health, hpv, cancer, awareness, https, free, sti, sexually* | *Even Disney Princesses Need HPV Shots, Cervical Cancer Screenings And STD Testing* |
| Comment | 1. Vaccine package inserts | *vaccine, know, hpv, study, insert, vaccines, understand, cdc, question, cause* | *Do you not know what the insert is? It only has premarketing information and it's a legal document. Current studies do prove the safety and efficacy of the vaccine* |
|  | 2. Vaccine risks | *vaccine, insert, risk, information, hpv, vaccines, doctor, read, ban, look* | *It's been tested in after market / post market settings as young as 9 years old since licencing.. pretty informally but enough to be allowed in package insert and prescribing info edits* |
|  | 3. User interactions within comment section | *vaccine, hpv, insert, cancer, good, people, post, com, understand, efa* | *Glad to see you giving residents good information. I'm sure you knew presenting the facts would generate some push back, but you did it anyway. Well done.* |
|  | 4. Scientific evidence on vaccines | *vaccine, hpv, study, report, cdc, link, insert, vaccines, evidence, read* | *The vaccine insert mentions the main clinical trials used to approve the vaccine, but not studies after.* |
|  | 5. Questioning information sources | *people, vaccine, read, source, hpv, look, time, data, vaccines, science* | *I would say to show me where you got this info. But, I'm not interested in reading a blog.* |

**Supplementary table 4.** Thematic topics, keywords, and examples Facebook posts and comments about the HPV vaccine from public groups.

|  | Topic | Keywords | Example |
| --- | --- | --- | --- |
| Post | 1. Covid-19 vaccine | *spl, virucidal, nasal, virus, sars, cov, covid, antiviral, activity, spray* | *Moderna is testing whether we have sterilizing immunity after the COVID19 vaccine… On the other hand, the HPV vaccine does prevent transmission.* |
|  | 2. Gardasil safety and death | *gardasil, go, government, vaccine, case, protect, win, couldn, die, drug* | *Gardasil Kills2* |
| Comment | 1. Vaccine allergic reactions | *vaccine, reaction, allergic, mask, wear, like, science, jab, create, get* | *An anaphylactic reaction is a severe allergic reaction. It can include things like hives and closing of airways making it difficult or impossible to breathe. Allergic reactions to vaccines, in general, are rare.* |
|  | 2. Mask wearing | *mask, time, kathleen, amatangelo, jones, wear, best, true, watch* | *Wear a mask after vaccination? So I dont give the virus to my vaccinated grandmother? Seems legit.* |
|  | 3. HPV vaccine injuries for children | *vaccine, ajalat, injury, jenniferã, mask, children, gardasil, case, program, compensation* | *HPV vaccine injuries are no longer extremely rare. In fact, neither are vaccine injuries in general.* |
|  | 4. Distrust in vaccine science | *people, time, tell, science, blind, things, close, truth, cancer, vaccine* | *The science to brainwash or the science of truth!! And also it does not take a scientist or dr to figure out the things that make absolute nonsense and the things that make sense!!*  *Sorry but do some honest to goodness research not blind faith and have a hard time hearing something different from what mainstream media is telling you!!* |
